# Asymmetric quantum correlations in the dynamical Casimir effect

**DOI:** 10.1038/s41598-019-45943-0

**Published:** 2019-07-02

**Authors:** Xue Zhang, Hui Liu, Zhihai Wang, Taiyu Zheng

**Affiliations:** 1 Center for Quantum Sciences and School of Physics, Northeast Normal University, Changchun, 130024 China; 2 Center for Advanced Optoelectronic Functional Materials Research, and Key Laboratory for UV-Emitting Materials and Technology of Ministry of Education, Northeast Normal University, Changchun, 130024 China

**Keywords:** Quantum information, Theoretical physics, Single photons and quantum effects

## Abstract

Considering the current available experimental studies on the dynamical Casimir effect (DCE) in superconducting microwave waveguides, we study asymmetric quantum correlations in microwave radiation. The asymmetric quantum correlations are created by the presence of detuning in the DCE. We study the asymmetric quantum steering and determine the parameter regions of one- and two-way quantum steering. It shows that steering from Bob to Alice is more difficult than steering from Alice to Bob. Moreover, we find regions that represent states that, although entangled, cannot be used for teleporting coherent states; however, the steerable states are appropriate for quantum teleportation. We investigate how the teleportation fidelity functions as an indicator of the quality of EPR steering in the DCE.

## Introduction

The dynamical Casimir effect (DCE) was theoretically predicted in 1970^1^. Pairs of particles created due to time-dependent external conditions can be achieved in very different setups, e.g., by a changing boundary condition^1,2^ or the index of refraction of the medium^3,4^. In recent years, considerable attention has been given to DCE by many physicists^5–11^. There is a vast body of literature on theoretical^5,6^ and experimental^7–11^ aspects of DCE.

In 2011, the DCE was first confirmed by an experiment constructed with a superconducting circuit terminated by a superconducting quantum interference device (SQUID)^11^. A more general revision about the DCE in the superconducting circuit has been reported in 2012^7^. The superconducting circuits are not only used in the experimental demonstration for the DCE, but also may be to verify the analogue Hawking radiation^7^ and to realize quantum computers. Moreover, in a array made of N superconducting open stripline waveguides, the entangled NOON states have been created^12^. The microwave radiation produced by the DCE in superconducting circuits therefore has high potential of being distinctly nonclassical. In experiments in 2011, pairs of photons generated by the DCE revealed quantum entanglement^13^ and quantum discord^14^. We will investigate the other useful forms of quantum correlations in the experiments.

On one hand, we focus on the asymmetric nature which is caused by the detuning in the DCE. We will employ Einstein-Podolsky-Rosen (EPR) steering^15–20^ to explore the asymmetric nature of the bipartite Gaussian system. In ref.^21^, EPR steering was discussed in continuous variable (CV) systems, but the effect of detuning was not taken into account. The asymmetric nature of the system is caused by detuning in the DCE, and the asymmetrical nonlocalities are receiving considerable attention^22–29^ for special tasks in quantum information processing, quantum metrology^30^, quantum state merging^31–33^, remote state preparation^34^ one-sided device-independent quantum key distribution^35^, and entanglement verification^36^. In contrast to symmetric systems, we show how one can produce a desired EPR state to fulfill a given directional quantum task by adding asymmetric amounts of detuning to each subsystem in the DCE. We also find the regions that represent one-way steerable states or two-way steerable states. On the other hand, we relate the entanglement and EPR steering to the teleportation fidelity^34,37,38^. We show that some states lying in the area are entangled, but they cannot be used as a quantum resource for teleportation. The teleportation fidelity is directly related to the strength of EPR steering. Therefore, the teleportation fidelity becomes an indicator of the quality of the EPR steering in the DCE.

The paper is organized as follows: In Sec. II, we review the DCE in superconducting circuits and derive the covariance matrix (CM) of the system. In Sec. III, we employ different entanglement markers in terms of the CM. We also include a detailed discussion on the relationship between the different entanglement markers and the teleportation fidelity for the DCE. We summarize the research in Sec. IV.

## DCE in Superconducting Waveguides

In 2011, an experimental observation of the DCE was reported^11^ in a superconducting waveguide (SW)^39,40^. The experiment demonstrated that the DCE produces a two-mode squeezed state^13,41–44^. The electromagnetic field in the SW can be characterized by the flux operator ${\rm{\Phi }}(x,t)$. Quantizing the electromagnetic field by the flux operator ${\rm{\Phi }}(x,t)$ and solving the massless, one-dimensional Klein-Gordon wave equation, ${\partial }_{xx}{\rm{\Phi }}(x,t)-{\upsilon }^{-2}{\partial }_{tt}{\rm{\Phi }}(x,t)=0$, the the flux operator ${\rm{\Phi }}(x,t)$ can be expressed in the form^13,39,40^

1 $${\rm{\Phi }}(x,t)=\sqrt{\frac{\hslash {S}_{0}}{4\pi }}\,\int \,\frac{d\omega }{\sqrt{\omega }}[{\hat{a}}_{\omega }{e}^{i(-\omega t+kx)}+{\hat{b}}_{\omega }{e}^{i(-\omega t-kx)}],$$

where ${\hat{a}}_{\omega }$ and ${\hat{b}}_{\omega }$ are the annihilation operators for incoming and outgoing photons of the SW, respectively. Here, *k* is the wave number, and *S*_0_ is the characteristic impedance. The DCE can be studied by the scattering theory. The time-dependent boundary condition brings about an effective length and mixes the independent incoming and outgoing modes. The effective length can be written in the form

2 $${L}_{{\rm{eff}}}(t)=\frac{{({{\rm{\Phi }}}_{0}/2\pi )}^{2}}{{E}_{J}(t){L}_{0}},$$

*L*_0_, ${{\rm{\Phi }}}_{0}=h$/$(2e)$ and ${E}_{J}(t)={E}_{J}[{{\rm{\Phi }}}_{{\rm{ext}}}(t)]$ represent the characteristic inductance per unit length of the waveguide, the magnetic flux quantum and the flux-dependent effective Josephson energy, respectively. ${{\rm{\Phi }}}_{{\rm{ext}}}(t)$ is the time-dependent applied magnetic flux through the SQUID. For a sinusoidal modulation with driving frequency ${\omega }_{d}$/$2\pi$ and normalized amplitude $\epsilon$, we obtain ${E}_{J}(t)={E}_{J}^{0}[1+\epsilon \,\sin \,{\omega }_{d}t]$ and an effective length modulation amplitude $\delta {L}_{{\rm{eff}}}=\epsilon {L}_{{\rm{eff}}}(0)$. In the perturbative regime, the outgoing modes are correlated with frequencies ${\omega }_{+}$, ${\omega }_{-}$ (${\omega }_{+}+{\omega }_{-}={\omega }_{d}$)^39^. In previous studies, the authors considered a symmetric situation by setting ${\omega }_{+}={\omega }_{-}={\omega }_{d}$/2. To investigate the effect of asymmetry, we introduce the detuning $\delta \omega$, by setting ${\omega }_{\pm }={\omega }_{d}$/$2\pm \delta \omega$. Noting ${a}_{\pm }=a({\omega }_{\pm })$ and ${b}_{\pm }=b({\omega }_{\pm })$, the relationship between the incoming and outgoing operators is written as

3 $${\hat{b}}_{\pm }=-\,{\hat{a}}_{\pm }-if{\hat{a}}_{\mp }^{+},$$

where

4 $$f=\frac{\delta {L}_{{\rm{eff}}}\sqrt{{\omega }_{+}{\omega }_{-}}}{\upsilon }=\frac{\epsilon {L}_{{\rm{eff}}}(0)\sqrt{{\omega }_{+}{\omega }_{-}}}{\upsilon }.$$

Let us consider the CM^6^ of the bipartite Gaussian CV system in the DCE. All the Gaussian properties can be obtained by the CM^14,21,45^

5 $${V}_{\alpha \beta }=\frac{1}{2}\langle {R}_{\alpha }{R}_{\beta }+{R}_{\beta }{R}_{\alpha }\rangle -\langle {R}_{\alpha }\rangle \langle {R}_{\beta }\rangle ,$$

where

6 $$R={({\hat{X}}_{-},{\hat{P}}_{-},{\hat{X}}_{+},{\hat{P}}_{+})}^{T},$$

is the vector of the field quadratures with elements:

7 $${\hat{X}}_{\pm }=({\hat{b}}_{\pm }+{\hat{b}}_{\pm }^{+})/\sqrt{2},{\hat{P}}_{\pm }=-\,i({\hat{b}}_{\pm }-{\hat{b}}_{\pm }^{+})/\sqrt{2}.$$

Similarly, the quadratures of the input fields are expressed as

8 $${\hat{X}}_{0\pm }=({\hat{a}}_{\pm }+{\hat{a}}_{\pm }^{+})/\sqrt{2},{\hat{P}}_{0\pm }=-\,i({\hat{a}}_{\pm }-{\hat{a}}_{\pm }^{+})/\sqrt{2}.$$

We suppose that the input fields are in a quasi-vacuum state confirmed by a small number of thermal photons ${n}_{+}^{{\rm{th}}}$, ${n}_{-}^{{\rm{th}}}$. Then, the CM of the input fields is

9 $${V}_{0}=(\begin{array}{llll}{n}_{0} & 0 & 0 & 0\\ 0 & {n}_{0} & 0 & 0\\ 0 & 0 & {m}_{0} & 0\\ 0 & 0 & 0 & {m}_{0}\end{array}),$$

The elements in Eq. (9) can be calculated.

10 $$\begin{array}{rcl}{n}_{0} & = & 1/2+{n}_{-}^{{\rm{th}}},\\ {m}_{0} & = & 1/2+{n}_{+}^{{\rm{th}}}.\end{array}$$

where ${n}_{\pm }^{{\rm{th}}}={({e}^{\tfrac{\hslash {\omega }_{\pm }}{kT}}-1)}^{-1}$, ${\omega }_{\pm }={\omega }_{d}$/$2\pm \delta \omega$ with the detuning $\delta \omega$. Similarly, we can obtain the CM of the output fields

11 $$V=(\begin{array}{llll}n & 0 & c & 0\\ 0 & n & 0 & -c\\ c & 0 & m & 0\\ 0 & -c & 0 & m\end{array}),$$

where

12 $$\begin{array}{rcl}n & = & 1/2+{n}_{-}^{{\rm{th}}}+{f}^{2}(1/2+{n}_{+}^{{\rm{th}}}),\\ m & = & 1/2+{n}_{+}^{{\rm{th}}}+{f}^{2}(1/2+{n}_{-}^{{\rm{th}}}),\\ c & = & f(1+{n}_{+}^{{\rm{th}}}+{n}_{-}^{{\rm{th}}}).\end{array}$$

## Asymmetric Quantum Correlations in the DCE

The DCE can produce microwave radiation, which exhibits non-local interaction properties, in the virtual particles of the quantum vacuum^13,14,21,45^. Therefore, the microwave radiation must possess quantum correlations. The theory of quantum correlations has been well developed in quantum optics, and the quantum nature of a Gaussian state can be test by many markers. In the following, we will discuss the Peres-Horodecki-Simon (PHS) criterion for entanglement, the EPR criterion for one- and two-way steering, and the teleportation fidelity in the DCE. We focus on two points: the asymmetric nature caused by the detuning in the DCE and how the teleportation fidelity works as an indicator in CV teleportation protocols.

### The PHS Criterion for Entanglement

Entanglement is the edge of quantum correlation. The PHS criterion has been proven to be the necessary and sufficient condition for the entanglement of bipartite Gaussian CV systems^46^, and it can be expressed simply in terms of the CM elements.

13 $${E}_{{\rm{NT}}}=({n}^{2}+{m}^{2}+2{c}^{2})-4{(nm-{c}^{2})}^{2} > \frac{1}{4}.$$

If Eq. (13) is satisfied, the bipartite Gaussian CV state will be entangled. We will use this PHS criterion to study the DCE radiation. By solving Eq. (13), we can get the range of *f* to ensure that the state is entangled. Taking a Taylor series expansion of *f*, ${n}_{+}^{{\rm{th}}}$ and ${n}_{-}^{{\rm{th}}}$, we obtain

14 $$f > \sqrt{\frac{2{n}_{+}^{{\rm{th}}}{n}_{-}^{{\rm{th}}}}{2+5({n}_{+}^{{\rm{th}}}+{n}_{-}^{{\rm{th}}})}}.$$

For the sake of a comparison with the results of ref.^45^, we consider no detuning. We can write ${\omega }_{+}={\omega }_{-}={\omega }_{d}$/2, and then ${n}_{+}^{{\rm{th}}}={n}_{-}^{{\rm{th}}}={n}^{{\rm{th}}}$. When ${n}^{{\rm{th}}}\ll 1$, the above inequality can be further simplified to $f > {n}^{{\rm{th}}}$. This result is consistent with the result reported in ref.^45^.

**Figure 1:**
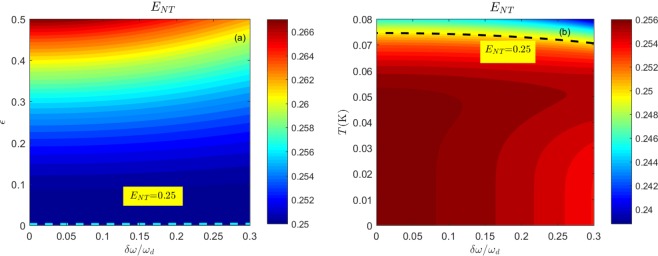
The PHS criterion for entanglement as a function of the detuning $\delta \omega$ and the driving amplitude $\epsilon$ for a temperature of *T* = 30 mK in (**a**) and the detuning $\delta \omega$ and the temperature for the driving amplitude $\epsilon =0.3$ in (**b**). The dashed lines in (**a**,**b**) represent ${E}_{NT}=0.25$, which is the critical value of the PHS criterion. The small parameter $f < 0.078$ is well within the perturbative analysis. The plots are realized with the experimental parameters ${\omega }_{d}=20\pi \,{\rm{GHz}}$, *L*_eff_(0) = 0.5 mm, and *v* = 1.2 × 10^8^ m/s.

The PHS criterion for the DCE is shown in Fig. 1. The figure visualizes the PHS criterion as a function of temperature, detuning, and driving amplitude. We observe that for a temperature of *T* = 30 mK, the PHS criterion is always larger than 1/4 for any sensible values of the driving amplitude and the detuning. In the case of a fixed $\epsilon =0.3$, we find that the entanglement vanishes at high temperatures within the given detuning regime, and the threshold value is almost 68 mK. We also observe that thermal noise tends to suppress entanglement, and the driving amplitude $\epsilon$ tends to promote entanglement.

### The EPR Criterion for Steering

Steering is a significant form of quantum correlations^47,48^ and is expressed asymmetrically between Alice and Bob. Asymmetric steering describes the idea of action at a distance of the EPR state, which allows Alice to adjust Bob’s state by means of local measurements^15^. On the operational side, the asymmetric steering can be useful for providing security in one-sided device-independent quantum key distribution schemes^35^. In this section, we focus on the asymmetric steering caused by the detuning condition in the DCE. We will employ EPR steering to explore the asymmetric nature of the bipartite Gaussian CV state. Mathematically, the EPR criterion can be calculated in terms of CM elements, that is, from the result of Q. Y. He^49^. The EPR steering criterion from Bob to Alice can be expressed by:

15 $${E}_{{\rm{B}}\to {\rm{A}}}=n-\frac{{c}^{2}}{m} < \frac{1}{2}.$$

We note that, being based on conditional variances, the EPR criterion is asymmetric under swapping subsystems. Then, the EPR steering criterion from Alice to Bob can be recast by exchanging *m* and *n* in Eq. (15),

16 $${E}_{{\rm{A}}\to {\rm{B}}}=m-\frac{{c}^{2}}{n} < \frac{1}{2}.$$

To satisfy the EPR steering criterion ${E}_{{\rm{B}}\to {\rm{A}}} < 1/2$ (or ${E}_{{\rm{A}}\to {\rm{B}}} < 1/2$) requires *f* to exceed the threshold value given by ${f}_{{\rm{B}}\to {\rm{A}}}$ (or ${f}_{{\rm{A}}\to {\rm{B}}}$). Solving the inequalities, we can obtain

17 $${f}_{{\rm{B}}\to {\rm{A}}} > \sqrt{\frac{2{n}_{-}^{{\rm{th}}}(1+2{n}_{+}^{{\rm{th}}})}{(2{n}_{-}^{{\rm{th}}}+1)\,(4{n}_{+}^{{\rm{th}}}+3)}},$$

18 $${f}_{{\rm{A}}\to {\rm{B}}} > \sqrt{\frac{2{n}_{+}^{{\rm{th}}}(1+2{n}_{-}^{{\rm{th}}})}{(2{n}_{+}^{{\rm{th}}}+1)\,(4{n}_{-}^{{\rm{th}}}+3)}}.$$

In the case of ${n}_{+}^{{\rm{th}}}={n}_{-}^{{\rm{th}}}={n}^{{\rm{th}}}$, the threshold values ${f}_{{\rm{B}}\to {\rm{A}}}$ and ${f}_{{\rm{A}}\to {\rm{B}}}$ are the same. We note that ${f}_{{\rm{B}}\to {\rm{A}}}={f}_{{\rm{A}}\to {\rm{B}}}=f > \sqrt{2{n}^{{\rm{th}}}/3}$ for ${n}^{{\rm{th}}}\ll 1$, which is consistent with the result reported in ref.^21^.

**Figure 2:**
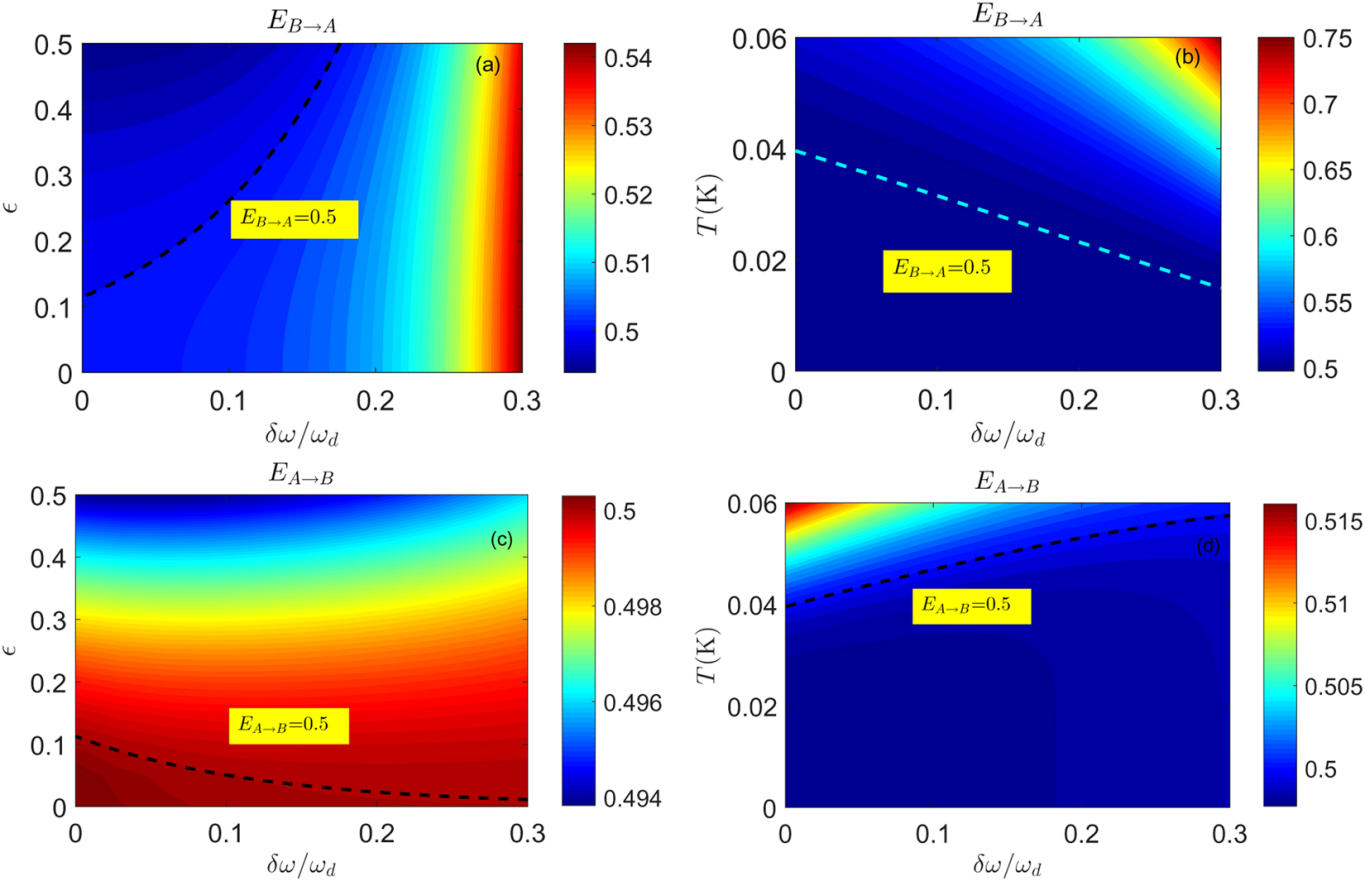
The EPR criterion for steering from B to A as a function of the detuning $\delta \omega$ and the driving amplitude $\epsilon$ in (**a**) and of the detuning $\delta \omega$ and the temperature *T* in (**b**). The dashed lines in (**a**,**b**) represent ${E}_{{\rm{A}}\to {\rm{B}}}=0.5$, which is the critical value of the EPR steering criterion from B to A. The EPR criterion for steering from A to B as a function of the detuning $\delta \omega$ and the driving amplitude $\epsilon$ in (**c**) and of the detuning $\delta \omega$ and the temperature *T* in (**d**). The dashed lines in (**c**,**d**) represent ${E}_{{\rm{A}}\to {\rm{B}}}=0.5$, which is the critical value of the EPR steering criterion from A to B. The small parameter $f < 0.078$ is well within the perturbative analysis. The parameters are the same as those in Fig. 1.

Figure 2 shows the behaviour of the EPR steering with respect to the driving amplitude, temperature and detuning. In contrast to symmetric systems (${\omega }_{+}={\omega }_{-}$), we introduce directional EPR steering to test the quantum correlations in the DCE. The presence of detuning causes asymmetric steering and makes steering from A to B easier than from B to A, as illustrated in Fig. 2. The sensitivity of the steering parameter ${E}_{{\rm{A}}\to {\rm{B}}}$ is asymmetrical, and one-way steering is evident. When both ${E}_{{\rm{B}}\to {\rm{A}}} < 1/2$ and ${E}_{{\rm{A}}\to {\rm{B}}} < 1/2$ are satisfied, two-way EPR steering is confirmed; i.e., the DCE state with $f > \,{\rm{\max }}\,\{{f}_{{\rm{B}}\to {\rm{A}}},{f}_{{\rm{A}}\to {\rm{B}}}\}$ can be used to produce two-way steering.

In Fig. 2(a), we observe that the EPR steering from B to A vanishes with the detuning $\delta \omega  > 0.18{\omega }_{d}$ for any driving amplitude at *T* = 30 mK. Figure 2(c) reveals that a certain value of the driving amplitude ($\epsilon  > 0.12$) is required to overcome the detuning and gives rise to EPR steering from A to B. We also find that thermal noise tends to suppress EPR steering from both ${E}_{{\rm{B}}\to {\rm{A}}}$ and ${E}_{{\rm{A}}\to {\rm{B}}}$ in Fig. 2(b,d), but in Fig. 2(c,d), we observe that EPR steering from A to B is highly robust to temperature and detuning.

### The Teleportation Fidelity in the DCE

In this section, we focus on how to evaluate the quality of entanglement in CV teleportation protocols. The PHS criterion is incapable of measuring entanglement in a quantitative manner. In contrast to the PHS criterion, the fidelity ${\mathcal F}$ of CV teleportation for a Gaussian state only depends on the entanglement quality of the resource itself. Accordingly, the fidelity ${\mathcal F}$ of teleportation is employed as a benchmark of an entangled state. Using the Gaussian state described by the CM in Eq. (11), ${\mathcal F}$ can be written as^50^

19 $$ {\mathcal F} ={(1+m+n-2c)}^{-1}.$$

Equation (19) shows that when ${\mathcal F} \le 1/2$, the teleportation protocol of a Gaussian state can be completed implemented with classical strategies; in contrary, if the resource is entangled, ${\mathcal F}$ will be larger than 1/2. Thus, the fidelity ${\mathcal F}$ is an indicator of the quality of the entanglement, and the state can be used as a quantum resource for teleportation. In other words, ${\mathcal F}  > 1/2$ requires *f* to exceed the threshold value $f > 1-{({n}_{+}^{{\rm{th}}}+{n}_{-}^{{\rm{th}}}+1)}^{-1/2}$. Considering no detuning condition, we can write ${n}_{+}^{{\rm{th}}}={n}_{-}^{{\rm{th}}}={n}^{{\rm{th}}}$. When ${n}^{{\rm{th}}}\ll 1$, the above inequality can be further simplified to $f > {n}^{{\rm{th}}}$. This result is the same with the result of the PHS criterion. From this perspective, the entangled states can be used for teleporting coherent states. However, considering the detuning condition, the threshold values are different of the fidelity and the PHS criterion. The entangled states are not appropriate for quantum teleportation. We can see that the detuning creates an important influence on the fidelity.

**Figure 3:**
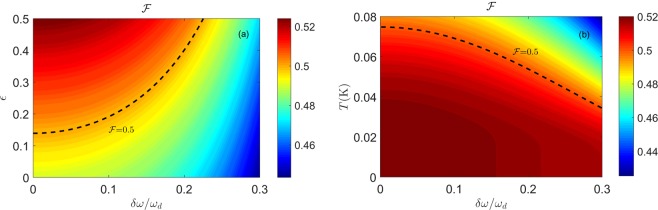
The teleportation fidelity as a function of the detuning $\delta \omega$ and the driving amplitude $\epsilon$ in (**a**) and of the detuning $\delta \omega$ and the temperature *T* in (**b**). The dashed lines in (**a**,**b**) represent ${\mathcal F} =0.5$, which is the critical value of the teleportation fidelity. The small parameter $f < 0.078$ is well within the perturbative analysis. The parameters are the same as those in Fig. 1.

In Fig. 3(a), we find that, in the case of $\delta \omega  > 0.226{\omega }_{d}$, the fidelity is always less than 0.5 for any driving amplitude. The fidelity is always less than 0.5 with $\epsilon  < 0.138$ for any detuning. In other words, below the region of the dashed line in Fig. 3(a), the states are not appropriate for the quantum teleportation. Figure 3(b) reveals that the fidelity is robust to detuning when the temperature is below 34 mK.

### Witnesses

By focusing on the DCE, we will employ four different witnesses to determine whether the states exhibit entanglement or steering.

20 $$\begin{array}{rcl}{w}_{{\rm{PHS}}} & = & 4{(nm-{c}^{2})}^{2}-({n}^{2}+{m}^{2}+2{c}^{2})+\frac{1}{4},\\ {w}_{{\rm{B}}\to {\rm{A}}} & = & n-\frac{{c}^{2}}{m}-\frac{1}{2},\\ {w}_{{\rm{A}}\to {\rm{B}}} & = & m-\frac{{c}^{2}}{n}-\frac{1}{2},\\ {w}_{ {\mathcal F} } & = & \frac{1}{2}-{(1+m+n-2c)}^{-1}.\end{array}$$

In summary, each witness corresponds to the above criteria [see Eqs (13), (15), (16) and (19)], which can be reduced to

21 $$\{\begin{array}{rcl}{w}_{{\rm{PHS}}} < 0 & \iff  & {\rm{entanglement}},\\ {w}_{{\rm{B}}\to {\rm{A}}} < 0 & \iff  & {\rm{steering}}\,({\rm{B}}\to {\rm{A}}),\\ {w}_{{\rm{A}}\to {\rm{B}}} < 0 & \iff  & {\rm{steering}}\,({\rm{A}}\to {\rm{B}}),\\ {w}_{ {\mathcal F} } < 0 & \Rightarrow  & {\rm{entanglement}}.\end{array}$$

The first three criteria are sufficient and necessary to confirm entanglement or steering for the DCE. The fourth criterion is only sufficient (not necessary) to confirm entanglement.

**Figure 4:**
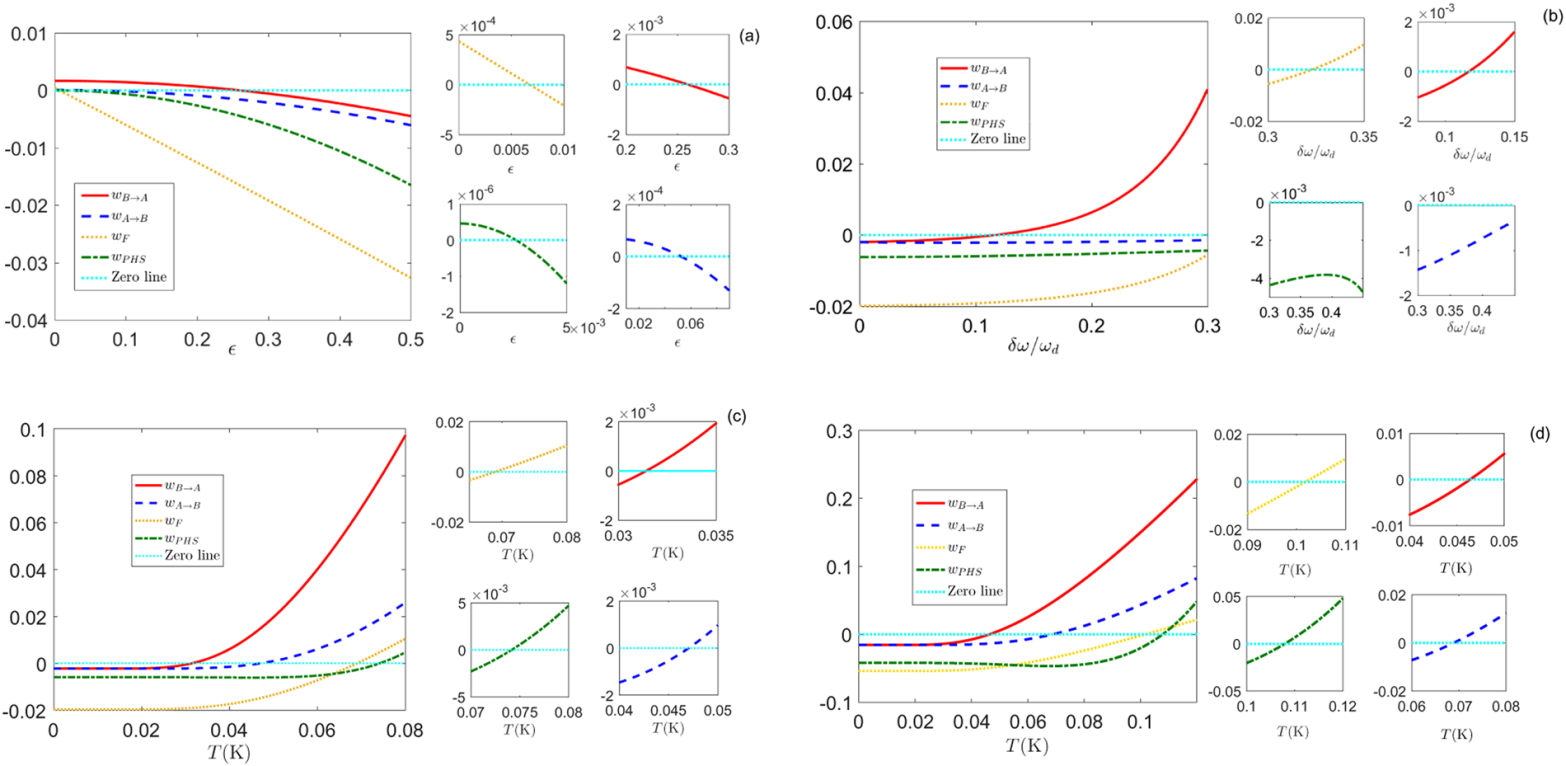
Plots of the different witnesses of Eq. (20) as entanglement markers. The witnesses as a function of the driving amplitude $\epsilon$ for a temperature of *T* = 30 mK in (**a**). The witnesses have threshold values of $\epsilon$ above which they are less than zero. The threshold values are $\epsilon \approx 0.0068$ for the teleportation fidelity, $\epsilon \approx 0.0027$ for entanglement, $\epsilon \approx 0.053$ for directional one-way steering from A to B, and $\epsilon \approx 0.26$ for directional one-way steering from B to A. In (**b**), the witnesses as a function of the detuning $\delta \omega$ for a driving amplitude $\epsilon =0.3$ and a temperature of *T* = 30 mK. The teleportation fidelity and steering from B to A vanish for the given detunings $\delta \omega \approx 0.33{\omega }_{d}$ and $\delta \omega \approx 0.11{\omega }_{d}$, respectively. However, the entanglement and steering from A to B do not disappear in this parameter regime. The witnesses as a function of the temperature *T* for (**c**) $\epsilon =0.3$ and (**d**) $\epsilon =0.8$. In (**c**), the witness of the teleportation fidelity is zero at $T\approx 69\,{\rm{mK}}$, and the entanglement vanishes at $T\approx 74\,{\rm{mK}}$. The temperature region of directional one-way steering from A to B is $31\,{\rm{mK}} < T < 47\,{\rm{mK}}$, and the region of two-way steering is $T < 31\,{\rm{mK}}$. In (**d**), the threshold temperature is replaced by $T\approx 107\,{\rm{mK}}$ for entanglement and $T\approx 101\,{\rm{mK}}$ for teleportation fidelity. The temperature regions of directional one-way steering from A to B and two-way steering are displaced to $47\,{\rm{mK}} < T < 68\,{\rm{mK}}$ and $T < 46\,{\rm{mK}}$, respectively. The parameters are: ${\omega }_{d}=20\pi \,{\rm{GHz}}$, *L*_eff_(0) = 0.5 mm, $\delta \omega =0.1{\omega }_{d}$, and *v* = 1.2 × 10^8^ m/s.

In Fig. 4 we compare the behaviours of the witnesses in Eq. (20) for realistic experimental parameters. We observe that the teleportation fidelity offers a more restrictive condition with respect to the PHS criterion in the given parameter regions. In other words, we find the regions representing states that, even if entangled, are not suitable for teleportation. From the perspective of teleportation, the above entanglement can be used for locally transforming the two subsystems, but it is not helpful for teleportation. If the states can be used as a quantum resource for teleportation, we will have ${w}_{ {\mathcal F} } < 0$. Interestingly, we also find that the EPR steering criterion is stricter than the teleportation fidelity criterion for any given value. If the states are steerable, the states will always be used for teleportation. The teleportation fidelity of the state is directly related to the strength of EPR steering.

Moreover, the presence of detuning creates asymmetric steering, making steering from B to A more difficult than from A to B, as illustrated in Fig. 4. We also find the parameter regions of directional one-way steering from A to B and two-way steering in this regime. In Fig. 4(b), the detuning also tends to suppress entanglement and steering, but entanglement and steering from A to B are almost insensitive to detuning in this regime. In Fig. 4(c,d), we find that thermal noise tends to suppress entanglement and steering, but the temperature threshold values of the four witnesses increase with the driving amplitude. For the driving amplitude $\epsilon =0.8$, the entanglement and teleportation fidelity are robust to thermal noise within 100 mK, and two-way steering is robust to thermal noise within 46 mK.

## Conclusions

In conclusion, we have analyzed asymmetric quantum correlations of the DCE in a superconducting circuit terminated by a SQUID. We studied the ability of the DCE to generate one- and two-way steering, forms of quantum correlation that are stronger than entanglement and essential for quantum technologies. We found the parameter regions of one- and two-way steering. The presence of detuning causes asymmetric steering, resulting in directional steering from Alice to Bob that is easier than from Bob to Alice. Moreover, we have theoretically investigated how the fidelity of CV teleportation of the DCE varies with the parameters. We observed regions that represent states that cannot be used for teleporting coherent states, although they are entangled. Interestingly, we also found that by fulfilling the EPR steering conditions, the states will be suitable for quantum teleportation, and the suitability is directly related to the strength of EPR steering. Finally, our results suggest that asymmetric correlations via detuning can be promising candidates for quantum tasks requiring a directional operation. An extension of our work can be exploited as a resource for quantum technologies.
